# Prostate cancer: net survival and cause-specific survival rates after multiple imputation

**DOI:** 10.1186/s12874-015-0048-4

**Published:** 2015-07-28

**Authors:** Adeline Morisot, Faïza Bessaoud, Paul Landais, Xavier Rébillard, Brigitte Trétarre, Jean-Pierre Daurès

**Affiliations:** University of Montpellier, Laboratory of Biostatistics, Epidemiology and Public Health (EA2415), 641, avenue du doyen Gaston Giraud, Montpellier Cedex 5, 34093 France; Hérault Cancer Registry, 208, rue des Apothicaires, Montpellier Cedex 5, 34298 France; Department of Urology - BeauSoleil Clinic, 119 avenue de Lodève, Montpellier, 34070 France

**Keywords:** Multiple imputation, Net survival, Cause-specific survival, ERSPC

## Abstract

**Background:**

Estimations of survival rates are diverse and the choice of the appropriate method depends on the context. Given the increasing interest in multiple imputation methods, we explored the interest of a multiple imputation approach in the estimation of cause-specific survival, when a subset of causes of death was observed.

**Methods:**

By using European Randomized Study of Screening for Prostate Cancer (ERSPC), 20 multiply imputed datasets were created and analyzed with a Multivariate Imputation by Chained Equation (MICE) algorithm. Then, cause-specific survival was estimated on each dataset with two methods: Kaplan-Meier and competing risks. The two pooled cause-specific survival and confidence intervals were obtained using Rubin’s rules after complementary log-log transformation. Net survival was estimated using Pohar-Perme’s estimator and was compared to pooled cause-specific survival. Finally, a sensitivity analysis was performed to test the robustness of our constructed multiple imputation model.

**Results:**

Cause-specific survival performed better than net survival, since this latter exceeded 100 % for almost the first 2 years of follow-up and after 9 years whereas the cause-specific survival decreased slowly and than stabilized at around 94 % at 9 years. Sensibility study results were satisfactory.

**Conclusions:**

On our basis of prostate cancer data, the results obtained by cause-specific survival after multiple imputation appeared to be better and more realistic than those obtained using net survival.

**Electronic supplementary material:**

The online version of this article (doi:10.1186/s12874-015-0048-4) contains supplementary material, which is available to authorized users.

## Background

In 2012, prostate cancer represented 28.5 % of all male incident cancers in France, with 56,841 new cases, far above lung or colorectal cancer, with 28,211 and 23,226 new cases, respectively [1]. In the early 2000s, the use of prostatic specific antigens (PSA) as a screening test led to a marked increase in the incidence of prostate cancer. It persisted up to 2005, and then declined, as reported in 2013 by Rébillard et al. [2]. As observed in the USA and certain European countries [1], the risk of presenting with prostate cancer during life is increasing, while the risk of death from prostate cancer is decreasing. Indeed, in 2012, prostate cancer was the third cause of death from cancer, (8876 deaths), which represented 10 % of all male cancer deaths. Net survival was 70 % and 90 % for cases diagnosed in 1990 and 2002, respectively [1].

In 2009, the incidence of prostate cancer was very low for patients aged under 50 years old and the median age at diagnosis of prostate cancer was 70 years old. It is thus a cancer of the elderly. In this context, competing risks of death are particularly important to be taken into account given the patient’s advanced age. Indeed, patients may die from causes other than prostate cancer. Thus, survival due to prostate cancer may be more difficult to estimate.

Estimations of survival rates are diverse and the choice of the appropriate method depends on the context. Moreover, obtaining the true cause of death is difficult in order to estimate cause-specific survival and it may be inaccurate. Some methods are very sensitive to the mortality rate of the general population which may be subject to significant variability.

Recently, the recommended method [3, 4] for estimating net survival (survival if the cause of death under consideration is the only cause of death) is Pohar-Perme’s estimator [5]. It is based on the mortality rate of the general population. Thus, for diseases such as prostate cancer, Pohar-Perme’s net survival can exceed 100 %, because population mortality tables are not representative of men presenting with prostate cancer. Moreover, Pohar-Perme’s method had been criticized by Dickman et al. [6], who showed that Pohar-Perme’s estimator may lead to “increased variability and lack of stability for long-term survival, particularly for older age groups”.

Given the increasing interest in multiple imputation methods, when several causes of death are observed and/or when a representative sample of causes of death is completed and validated by experts, we explored the interest of a multiple imputation approach to estimate cause-specific survival.

The use of multiple imputation in survival analysis is now widespread. However, Goetghebeur and Ryan [7], Andersen et al. [8], Lu and Tsiatis [9], Gao and Tsiatis [10], Lu and Liang [11], Bakoyannis et al. [12] and Sen et al. [13] all proposed methods for estimating regression parameters, but not for estimating survival function. Only a few authors have proposed methods to estimate cumulative incidence, see for example Lee et al. [14, 15], Nicolaie et al. [16] or Moreno-Betancur and Latouche [17].

Our aim was to compare three measures:

- the results of Pohar-Perme’s net survival based on the database without causes of death
- the pooled cause-specific survival of Kaplan-Meier [18] after multiple imputation, when censoring other-cause deaths
- the pooled cause-specific cumulative incidence estimator after multiple imputation, accounting for deaths due to other causes as competing risks.

In the next section, European Randomized Study of Screening for Prostate Cancer (ERSPC) database and statistical methods are described. In Section ‘Results’, the constructed multiple imputation model and the 20 multiply imputed datasets created are presented as well as cause-specific and net survival. Sensitivity analyses results devoted to testing the robustness of our model are also exposed. A discussion concludes the paper.

## Methods

### ERSPC database

This study included 2844 men, aged between 56 and 78 years old, presenting with a prostate cancer and included in the ERSPC [19–21] from 2003 to 2011, in the Hérault department, France. The clinical variables were completed from the Hérault cancer register, and the causes of death from the ERSPC.

The following clinical variables were recorded: PSA level at diagnosis (ng/ml), clinical staging (cT,cN,cM) based on tumour size, regional lymph nodes and metastasis, type of first treatment, Gleason score, PSA level post treatment (ng/ml) and pathological staging (pT,pN,pM). Comorbidities and residual tumour after surgery were not taken into account as these data had not been collected for the whole study.

Clinical tumour stages (cTstage) were categorized as 1a,1b,1c for a tumour present but not detectable clinically or with imaging, 2a,2b,2c for a circumscribed tumour in the prostate tissue and stages 3a, 3b and 4 when the tumour had invaded the capsule or other nearby structures. Clinical N and M stages were coded 0/1 for absence/presence, respectively. Since the pathological stagings pT and pN are available only when a surgical procedure is performed, we included 2 new variables labelled T _*new*_ and N _*new*_ that are equal to pT, pN, respectively provided that the first treatment is surgery (we did not consider other surgical treatment), and these were equal to cT, cN, otherwise. Note that we always have cM=pM because there are very few biopsies.

The Gleason score was used to evaluate the prognosis of prostate cancer. This score is based on the degree of differentiation of the tumour. The score was taken from Gleason biopsy, not collected from Gleason prostatectomy. This score is the sum of the two grades (ranging from 1 to 5) most often represented in the analysed tumour. It ranges from 2 to 10. Gleason scores of 2 to 6 were categorized as “low risk”, Gleason 7 as “intermediate risk” and Gleason 8 to 10 as “high risk”.

The first treatment was coded as surgery, hormone therapy, chemotherapy, radiotherapy, high intensity focused ultrasound (HIFU) or surveillance. In order to form large enough groups of patients with the same level of risk, we grouped together those who were undergoing hormone therapy and chemotherapy and also those under surveillance and HIFU. Note that surveillance includes both watchful waiting and active surveillance.

We also used the d’Amico [22] generalized score built on the PSA level at the onset of diagnosis, the clinical stage T,N,M and the Gleason score. Four groups were formed as described in the Additional file 1.

To analyse the effect of age at diagnosis, PSA level at diagnosis and PSA level after treatment of prostate cancer, we considered these variables in a continuous form. Note that the expected PSA level after treatment varies according to the treatment.

All variables were categorized according to urologists opinion.

For the patients who died, information on their dates of death was obtained from the National Directory for the Identification of Natural Persons (RNIPP). The RNIPP identifies the vital status in France.

Causes of death were obtained from the CépiDc (French epidemiology centre of the medical causes of death) for the patients who had died before December 31st, 2010. The causes of death for all patients who died after December 31st, 2010 were missing because the request had not yet been made to CépiDC. Among the patients who died (322), patients with prostate cancer as cause of death (53 patients) and prostate cancer mentioned in part 1 of death certificate (2 patients) were considered died of prostate cancer. Other patients with observed cause of death (106 patients) were considered dying of other causes. Consequently there are 161 patients (50 %) with cause of death missing.

Follow-up was performed up to June 30, 2013 (end point date). The duration of follow-up was defined as the time elapsed between diagnosis and death if the patient had died, and the date of last news if the patient was lost from follow-up, and the censoring date otherwise. Forty-six patients were excluded because their dates of last news was the same as the date of diagnosis.

The shortest follow-up time was 1 day, and the longest almost 10 years (3624 days). The mean follow-up time was 1822 days (almost 5 years) and the median 1853 days (slightly more than 5 years).

Data used in this study are publicly available and approved by two ethical committees for studies using human subjects (National Data Processing Consultative Committee for Medical Research-CCTIRS- and by the Commission Nationale Informatique et Libertés-CNIL) which provided approval to access at population-based cancer, ERSPC and RNIPP data in Hérault and advocates that all medical information are confidential and anonymous (declaration n° 900075).

### MAR hypothesis

Missing data mechanism, which is the process that governs the probability of being missing, can be classified into three categories [23]:

- Missing Completely At Random (MCAR): the probability of missingness depends neither on the observed data nor on missing data.
- Missing At Random (MAR): the probability of missingness may depends on the observed data but not on missing data values.
- Missing Not At Random (MNAR): the probability of missingness depends on the observed data and on missing data values.

From these, the MAR assumption is a starting point in multiple imputation since MICE performs well when it holds. We examined all the variables to be imputed and explained the reason of missingness. As MAR hypothesis is essential, it is important to assess it, especially for the cause of death variable that has 50 % of missing values. Even if they are not widely used and their practical value is unclear, Enders [24] proposed 2 tests to assess MCAR versus MAR. Note that it is impossible to test MNAR versus MAR because one would need missing information. The first method, proposed by Dixon [25], uses a series of independent *t*-tests to compare missing data subgroups, and the second one, by Little [26], uses a multivariate extension of the t-test approach.

Dixon’s test was performed on all the variables and, in particular, for the cause of death. This approach separates the missing and the complete cases on a particular variable and uses a *t*-test to examine group mean differences on other variables in the data set. The MCAR mechanism implies that the cases with observed data should be the same as the cases with missing values, on average. Therefore, if all the tests were non-significant, data were considered as MCAR; otherwise, a significant test suggested that the data were MAR or MNAR [24].

For quantitative variables (age at diagnosis, PSA level at diagnosis and PSA level after treatment) a Mann-Whitney-Wilcoxon test was used to explore whether the means were equal between the subgroups of observed versus non-observed variables tested. For qualitative variables (First treatment, cTstage, cNstage, cMstage, Gleason, pTstage, pNstage) the Fisher’s exact test was performed.

Moreover, distributions of patient’s characteristics were compared between completed causes of death and missing causes of death.

### Multiple imputation method and model

We used multiple imputation by chained equation [27, 28] to create 20 multiply imputed datasets. Incomplete variables were imputed under fully conditional specification [29] because of its flexibility to specify the method and the set of predictors to be used for each incomplete variable. Calculations were made in R 3.0.2 [30] using the mice package [31].

The main variable of interest to impute was the variable cause of death, with 50 % missing data. All variables available and the related number of missing values are displayed in Table 1. They were all imputed; 4,961 out of 23,272 records (21.3 %) were incomplete.

**Table 1:** Number of missing values for the variables of interest

Variable	Size	Missing data	Perc.
Cause of death	Deceased: 322	161	50 %
PSA at diagnosis	All: 2844	177	6.2 %
cT stage		589	20.7 %
cN stage		1815	63.8 %
cM stage		386	13.6 %
First treat.		292	10.3 %
Gleason		78	14.6 %
PSA after treat.		770	27.1 %
pT stage	Those who had surgery: 1521	92	6 %
pN stage		601	39.5 %

*Perc*. percentage, *treat*. treatment, *diag*. diagnosis

PSA at diagnosis, cTstage, cNstage, cMstage, Gleason, first treatment, and PSA after treatment were imputed with the default method in mice. Consequently, PSA at diagnosis and PSA after treatment that are continuous variables were imputed with a predictive mean matching method [26]. cNstage and cMstage were imputed with a logistic regression method since it is a binary variable. cTstage, Gleason and first treatment are categorical variables with more than 2 unordered categories, so they were imputed under a multinomial logit model.

Cause of death was imputed with a logistic regression method only if the patient was dead. Variables pNstage and pTstage were imputed with a logistic regression method and multinomial logit method, respectively, only if there had been a surgical procedure. For cause of death, pNstage and pTstage, special imputation functions were created.

As derived variables, we had T _*new*_ and N _*new*_ (=cTstage and cNstage, respectively, if no surgery procedure had been performed and pTstage and pNstage, respectively, if a surgical procedure had occurred) and d’Amico score calculated from Gleason, PSA at diagnosis and cTstage.

To preserve the relationships in the data and the uncertainty about these relationships we used a predictor matrix created by quickpred [32]. The quickpred function calculates correlations between variables and the proportion of usable cases and combines them automatically in a matrix [31]. Moreover, it is possible to specify the minimum correlation and the proportion of usable cases. As a starting predictor matrix, we defined the minimum proportion of usable cases is at least 0.4 and the minimum correlation is at least 0.1. Then, the clinicians specified the set of predictors to be used for each variable to impute and validated the predictor matrix.

Otherwise, since the complete-data model is a survival model, we explored the interest of adding the event indicator and the Nelson-Aalen estimator of the cumulative hazard to the survival time, H(T), in the imputation model as recommended by White and Royston [33]. The correlation between H(T) and T was calculated and was equal to 0.998. So, for these data, it matters little whether we took H(T) or T as predictor [32]. Then, the correlations between the variables to impute and H(T), T and event indicator were calculated. As correlations don’t exceed 0.2, two multiple imputation with and without time and event indicator as predictors were performed, and we compared the survival analysis. There were no differences between the curves, so we decided not to take into account time and event indicator in the imputation model. The prediction matrix used in this imputation model is presented in Table 2.

**Table 2:** Predictor matrix

	Cause of death	Age	PSA diag.	cT	cN	cM	First treat.	Gleason	pT	pN	PSA after treat.	T _*new*_	N _*new*_	d’Amico
Cause of death	0	1	1	0	0	1	1	1	0	0	1	1	1	1
Age	0	0	0	0	0	0	0	0	0	0	0	0	0	0
PSA diag.	0	1	0	1	1	1	0	1	0	0	0	0	0	0
cT	0	1	1	0	0	0	0	1	0	0	0	0	0	0
cN	0	1	1	1	0	1	0	1	0	0	1	1	0	1
cM	0	1	1	1	0	0	0	1	0	0	0	0	0	0
First treat.	0	1	1	1	1	1	0	1	0	0	0	0	0	1
Gleason	0	1	1	0	1	1	1	0	0	0	0	1	0	0
pT	0	1	0	1	0	1	1	0	0	0	0	0	0	0
pN	0	1	0	1	0	1	1	0	0	0	0	0	0	0
PSA after treat.	0	1	1	0	0	1	1	0	0	0	0	1	0	0
T _*new*_	0	0	0	0	0	0	0	0	0	0	0	0	0	0
N _*new*_	0	0	0	0	0	0	0	0	0	0	0	0	0	0
d’Amico	0	0	0	0	0	0	0	0	0	0	0	0	0	0

The rows correspond to variables to impute and the columns to the predictor. A 1 indicates that the column variable is used as a predictor to impute the row variable *Diag*. diagnosis, *treat*. treatment

A visiting scheme was specified to choose an imputation order. Theoretically, the visiting scheme is irrelevant as long as each column is visited often enough [31], but to be more efficient, a clinical chronological order was chosen: PSA at diagnosis, cTstage, cNstage, cMstage, Gleason, first treatment, pTstage, pNstage, PSA after treatment and cause of death.

Finally, Brand and Van Buuren [32, 34] have shown that MICE algorithm can converge with just 5 iterations. However, as some applications can require more iterations and as computations are not tedious, we set the number of iterations for this imputation model at 20.

Multiple imputation with *m*=10, 20 and 30 multiply imputed datasets were performed. Mean relative efficiencies for Kaplan-Meier estimator were calculated on each *m* and were equal to 0.97, 0.99 and 0.99, respectively. Survival analysis were compared on multiple imputation with 10, 20 and with 30 multiply datasets, there were no differences between the curves and between the confidence intervals. Since it is recommended to set the number of multiple imputations to the average of missing data [35] and there were 21 % of missing values, *m*=20 datasets were kept.

### Survival analysis

Three survival estimators were compared: Pohar Perme’s estimator, Kaplan-Meier estimator and cause-specific cumulative incidence estimator.

Pohar-Perme’s net survival was estimated on the original data base without imputation. This method does not require the causes of death: it uses the mortality rates of the general population to estimate the survival. Pohar-Perme’s net survival was calculated with the function rs.surv of relsurv package [36].

The next step was to analyse the 20 multiply imputed datasets with a cause-specific survival approach. Cause-specific survival was firstly estimated using Kaplan-Meier estimator when censoring other-cause deaths on each dataset separately, with the survfit function of survival package [37]. However, the cause-specific estimates that are based on the imputation model can be interpretable as estimates of net survival only if it is reasonable to assume independence between prostate cancer death and death from other causes than prostate cancer. This assumption could hold if the causes of death are correctly assigned.

Therefore, competing risks survival analysis was estimated accounting for death due to other causes as competing risks, with the cuminc function of cmprsk package [38].

### Rubin’s rules after complementary log-log transformation

The 20 Kaplan-Meier survivals and the 20th competing risks survivals were pooled using Rubin’s rules after complementary log-log transformation [39] to obtain the two pooled cause-specific survival and their confidence interval. As far as we know, Rubin’s rules after complementary log-log transformation were never presented in the literature.

In the first paragraph, Rubin’s rules [40] are remembered and then Rubin’s rules after complementary log-log transformation are presented.

#### Rubin’s rules [40]:

Let *Q* be the parameter of interest. After the multiple imputation, we had $\hat {Q}_{i}$, *i*=1,...,*m* wherein *m* was the number of multiple imputations, and *U*_*i*_, *i*=1,...,*m* the estimated variance for each imputed data set.

Rubin defined the pooled parameter of interest as:

(1) $$\begin{array}{@{}rcl@{}} \bar{Q}=\frac{1}{m}\sum\limits_{i=1}^{m}\hat{Q}_{i} \end{array} $$

And the total variance for this estimate was

(2) $$\begin{array}{@{}rcl@{}} T= \bar{U} + \left(1+ \frac{1}{m} \right)B \end{array} $$

in which

(3) $$\begin{array}{@{}rcl@{}} \bar{U}=\frac{1}{m}\sum\limits_{i=1}^{m}U_{i} \end{array} $$

was the pooled variance and

(4) $$\begin{array}{@{}rcl@{}} B=\frac{1}{m-1}\sum_{i=1}^{m}\left(\hat{Q}_{i}-\bar{Q}\right)^{2} \end{array} $$

was the between-imputation variance.

#### Rubin’s rules after complementary log-log transformation:

In our study, the parameters of interest are the survival probabilities and their confidence intervals.

For each imputed data set, at each time *t*_*j*_, *j*=1,...,*J*, we obtain the survival probabilities $\hat {S}_{i}(t_{j})$, *i*=1,...,*m* and their variance $V[\!\hat {S}_{i}(t_{j})]$, *i*=1,...,*m*.

According to Marshall et al. [39], the correct way of combining survival probabilities is to use Rubin’s rules after complementary log-log transformation.

For *i*=1,...,*m* and *j*=1,...,*J*, we define

$$\hat{Q}_{i}(t_{j})=log\left(-log\left[1-\hat S_{i}(t_{j})\right]\right) $$

So by applying the equation (1) of Rubin’s rules we obtain ∀*t*_*j*_, *j*=1,...,*J*:

$$\bar{Q}(t_{j})=\frac{1}{m} \sum\limits_{i=1}^{m} \hat Q_{i}(t_{j}) = \frac{1}{m} \sum\limits_{i=1}^{m}log\left(-log\left[1-\hat S_{i}(t_{j})\right]\right) $$

We also define, for *i*=1,...,*m* and *j*=1,...,*J*

$$U_{i}(t_{j})= Var(\hat{Q}(t_{j})) = Var\left[log\left(-log\left[1-\hat S_{i}(t_{j})\right]\right)\right] $$

In order to obtain *U*_*i*_(*t*_*j*_) depending on $V[\!\hat {S}_{i}(t_{j})]$, we use the *δ*-method [41]:

Using *δ*-method with $g(\hat {S}_{i}(t_{j}))=log(-log(1-\hat {S}_{i}(t_{j})))$, we obtain:

$$\begin{aligned} {}Var &\left[log(-log[\!1-\hat S_{i}(t_{j})])\right] \\&\approx \left(\frac{-1}{log \left[1-\hat S_{i}(t_{j}) \right] \times \left[1-\hat S_{i}(t_{j})\right]} \right)^{2} \times Var\left[\hat S_{i}(t_{j})\right] \end{aligned} $$

And finally,

$$U_{i}(t_{j}) \approx \frac{Var\left[\hat S_{i}(t_{j})\right]}{\left[ log (1-\hat S_{i}(t_{j})) \times (1-\hat S_{i}(t_{j})) \right]^{2}} $$

According to equations (3) and (4), we have:

$${}\bar U(t_{j}) \,=\, \frac{1}{m} \sum\limits_{i=1}^{m} U_{i}(t_{j}) \!\approx \!\frac{1}{m} \sum\limits_{i=1}^{m} \frac{Var\left[\hat S_{i}(t_{j})\right]}{\left[\! log (1\,-\,\hat S_{i}(t_{j}))\! \times\! (1-\hat S_{i}(t_{j}))\! \right]^{2}} $$

$${}B(t_{j}) = \frac{1}{m-1} \sum\limits_{i=1}^{m} \left[ \hat Q_{i}(t_{j}) - \bar Q(t_{j}) \right]^{2} $$

And using equation (2) the total variance for $\bar {Q}$ is:

$$T(t_{j}) = \bar{U}(t_{j}) + \left(1+\frac{1}{m}\right) B(t_{j}) $$

By definition, the 95 % confidence interval is:

$$\left[\bar{Q}(t_{j}) - 1.96 \sqrt{T(t_{j})} ; \bar{Q}(t_{j}) + 1.96 \sqrt{T(t_{j})} \right] $$

The pooled survival probabilities and the pooled confidence interval at each time *t*_*j*_, *j*=1,...,*J* must then be back transformed by 1− exp(− exp()), and we obtain:

(5) $$\begin{array}{@{}rcl@{}} \bar{S}(t_{j}) &=& 1 - \exp(-\exp (\bar{Q}(t_{j}))) \end{array} $$

(6) $$\begin{array}{@{}rcl@{}} {CI}_{95}(t_{j}) &=& \left[1 - \exp(-\exp (\bar{Q}(t_{j}) \pm 1.96 \sqrt{T(t_{j})}) \right] \end{array} $$

### Sensitivity analysis

#### First method

We tested our multiple imputation model on a complete dataset, from which we randomly removed different percentages of causes of death. For this, we created a sub database with all the 161 dead patients with a known cause of death (on the 322 initially deceased patients) and with half of patients still alive randomly selected, in order to have a proportion of dead/alive patients similar to the one for the whole database.

Thereby, the created sub-database was composed of 1422 patients, 161 of whom had died and a cause of death was identifiable. We could thus calculate the true cause-specific survival. Of these 161 patients, 10 %, 30 %, 50 %, 70 % and 90 % of causes of death were randomly removed and the variables were imputed with the same imputation model as before. As the variables were randomly removed, MCAR hypothesis holds. For each case, we compared the true cause-specific survival with the pooled cause-specific survival after the imputations of causes of death had been performed.

#### Second method

In order to test our imputation model, we also considered the 20 first multiply imputed datasets from the original data (2844 patients). For each imputed dataset, the 161 original causes of death present in the original data (106 deaths from other causes and 55 cancer deaths) were deleted, and the 161 imputed causes of death were retained. Thus, the MAR assumption still holds if the original missingness mechanism is MAR. Then, the 161 deleted causes of death were imputed using the same imputation model as before. Twenty new multiply imputed datasets were created, for each first multiply imputed dataset.

#### Third method

The last sensitivity analysis was conducted by cross-validation according to Gelman et al. [42]. The first 20 multiply imputed datasets were considered, as used above in the second method. However, instead of removing all the 161 original causes of death present in the original data set, 161 causes of death were randomly removed from these imputed datasets and the missing data were re-imputed using the same imputation model. For each of the first 20 multiply datasets the true and pooled cause-specific survival were compared.

## Results

### MAR hypothesis

For the main variable of interest, i.e. cause of death, among the 322 patients who died, 170 had died before December 31st, 2010 and 152 had died after December 31st, 2010. Among the 170 patients who had died before December 31st, 2010, there were 9 missing causes of death. The causes were missing because the CépiDC did not find these patients (e.g. moving abroad). Among the 152 patients who had died after December 31st, 2010, there were 152 missing causes of death, because the request had not been made to the CépiDC. So, the causes of death seemed to be MAR.

For the other variables, the reasons for missingness were due to incomplete medical files, or errors of data entry, so they seemed to be MAR.

The Dixon’s tests [25] were performed for all the variables to imputed. For each variable, a binary missing data indicator was created. Independent Mann-Whitney-Wilcoxon tests and Fisher’s exact tests were used to assess difference between the two groups created by the indicator on quantitative and qualitative variables, respectively.

For the variable cause of death, the Mann-Whitney-Wilcoxon tests for age at diagnosis, PSA level at diagnosis and PSA level after treatment were not statistically significant, p-values were equal to 0.98, 0.61 and 0.14, respectively. The Fisher’s exact tests for cTstage, cNstage, cMstage, first treatment, Gleason, pTstage and pNstage were also not statistically significant (taking into account repeat testing) with p-values equal to 0.37, 0.78, 0.13, 0.60, 0.04, 0.81 and 0.57, respectively. It thus indicated that the causes of death were MCAR.

On other variables to impute, Dixon’s tests were statistically significant for at least one variable. This means that other variables to imputed were not MCAR, but were MAR (as suspected above) or MNAR.

Moreover, distribution of patient’s characteristics was compared for completed and missing causes of death. The distribution was similar for completed and missing causes of death, see Table 3. So for all variables to impute, we assumed that the data were MAR.

**Table 3:** Distribution of baseline covariates for completed and missing causes of death and for all the patients

	Observed			Missing			*χ* ^2^		Total (*N*=2844)	
	cause of death			cause of death						
	(*n*=161)			(*n*=161)						
	No. of			No. of					No. of	
	patients^a^	%		patients^a^	%		P-value		patients	%
Age, years							0.82			
≤ 70	102	63		99	61				1931	68
> 70	59	37		62	39				913	32
PSA at diagnosis, ng/ml							0.62			
Missing	28	17		14	9				177	6
≤ 10	70	44		72	45				1952	69
> 10	63	39		75	46				715	25
cT stage							0.37			
Missing	38	24		21	13				589	21
cT1	58	36		52	32				1430	50
cT2a	15	9		17	11				243	9
cT2b	8	5		13	8				147	5
cT2c	19	12		20	12				212	7
cT3/cT4	23	14		38	24				223	8
cN stage							0.95			
Missing	138	86		112	70				1815	64
0	16	10		36	22				978	34
1	7	4		13	8				51	2
cM stage							0.13			
Missing	35	22		28	17				386	14
0	87	54		104	65				2355	2355
1	39	24		29	18				103	4
First treatment							0.92			
Missing	28	17		20	12				292	10
Surg.	51	32		51	32				1521	53
Radio.	23	14		28	17				467	16
Surv.+HIFU	5	3		4	2				111	5
Horm.+chemo.	54	34		58	36				453	16
Gleason							0.02			
Missing	14	9		8	5				78	3
2-6	68	42		50	31				1393	49
7	42	26		66	41				1150	40
8-10	37	23		37	23				223	8
d’Amico generalized							0.26			
Missing	27	17		7	4				291	10
Low	27	17		23	14				800	28
Intermediate	35	21		49	30				1136	40
High+Locally adv.	32	20		46	29				483	17
*N* ^+^ + *M* ^+^	40	25		36	22				134	5
pT stage							0.80			
Missing	116	72		117	73				1402	49
pT2a	10	6		11	7				280	10
pT2b	13	8		9	5				314	11
pT2c	14	9		14	9				619	22
pT3/pT4	8	5		10	6				229	8
pN stage							0.69			
Missing	130	81		123	77				1911	67
0	25	15		28	17				891	31
1	6	4		10	6				42	2
PSA after treat., ng/ml							0.68			
Missing	72	45		55	34				770	27
≤ 0.07	26	16		35	22				1145	40
> 0.07	63	39		71	44				929	33

*Surg*. surgery, *radio*. radiotherapy, *surv*, surveillance, *Horm*, Hormone therapy, *chemo*. chemotherapy, *inter*, intermediate, *treat*, treatment ^a^Number of deceased patients

### Imputation model convergence and diagnostics.

Multiple imputation convergence per variable is shown in the Additional file 2. The streams are freely intermingled with each other, without showing any definite trends [32], so the convergence is diagnosed. Moreover, we calculated the $\hat {R}$ statistic [43]. If it is smaller than 1.1 (i.e. the difference of the within and between-variance is trivial), the imputation is considered as convergent [44]. As we can see on Table 4, all the $\hat {R}$ statistics are smaller than 1.1, indicating the imputation convergence.

**Table 4:** $\hat {R}$ statistics of imputed variables

Var.	Cause of	PSA at	cT	cN	cM	First	Gleason	pT	pN	PSA after treat.
	death	diag.				treat				
$\hat {R}$	1.04	0.99	1.01	1.07	1	0.99	1.02	0.99	1	0.99

*diag*. diagnosis, *treat*. treatment

Diagnostics for multiple imputation models consist in evaluating the difference between observed and imputed data. For the two quantitative variables, PSA at diagnosis and PSA after treatment, distributions of observed and imputed data were similar. Qualitative variables were also compared for observed and imputed values.

For the main variable of interest, cause of death, 161 values were observed and 161 were imputed. Out of the 161 values observed, 106 (65.8 %) of them were “other causes” and 55 (34.2 %) were “prostate cancer”. Considering the 20 imputed values of “other causes” and “prostate cancer”, minimum values were equal to 43 and 96, respectively, maximum values were equal to 65 and 118, means were equal to 52.90 (32.9 %) and 108.1 (67.1 %) and medians were equal to 53 and 108, respectively. *χ*^2^ tests were performed to test the differences between imputed values and observed values for the means, minimum and maximum values. The p-values were equal to 0.89, 0.63 and 0.88, respectively. Percentages of observed and imputed values were also similar for the other qualitative variables. We therefore concluded that the imputations could be used to complete the missing data for the variables involved.

### Survival analysis

Table 5 displays the overall, net and pooled cause-specific survivals of Kaplan-Meier and competing risks rates (%) at 1, 3, 5 and 10 years, together with their confidence intervals. The results were satisfactory for the two pooled cause-specific survival rates after MI, but not for net survival (1.01 % at 1 year and 10 years).

**Table 5:** Overall survival, net survival and cause-specific survivals of Kaplan-Meier and competing risks after multiple imputation with their confidence intervals

	Overall	Net	Pooled Kaplan-Meier	Pooled competing risks
1 year	0.98 [0.98-0.99]	1.01 [1-1.01]	0.995 [0.992-0.997]	0.995 [0.991-0.997]
3 years	0.94 [0.93-0.95]	0.99 [0.99-1]	0.975 [0.968-0.981]	0.976 [0.969-0.981]
5 years	0.89 [0.88-0.91]	0.99 [0.98-1]	0.962 [0.952-0.970]	0.963 [0.954-0.971]
10 years	0.81 [0.78-0.83]	1.01 [0.98-1]	0.937 [0.915-0.955]	0.941 [0.921-0.957]

**Fig. 1:**
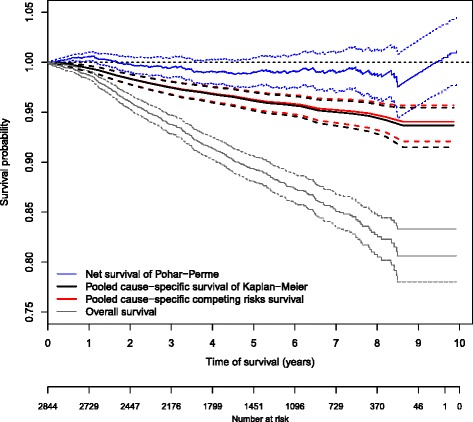
Overall, net, and pooled cause-specific survival curves and their confidence intervals

Figure 1 shows the overall, net, and the two pooled cause-specific survival curves. The net survival exceeded 100 % for almost the first 2 years of follow-up, then it decreased and was finally still greater than 100 % after 9 years. The pooled cause-specific survival using the Kaplan-Meier estimator decreased slowly and then stabilized at around 94 % at 9 years of follow-up. This is probably because the likelihood of death from other causes was higher than the likelihood of death from cancer, particularly for aged patients, and because the mortality rates for men in the general population are not representative of men with prostate cancer. The pooled cause-specific survival using competing risks method was almost equal to that of Kaplan-Meier estimator.

Table 6 displays overall, net and pooled cause-specific survivals of Kaplan-Meier and competing risks (%) at 1, 3, 5 and 10 years and their confidence intervals according to age. The results were similar to Table 5, the two pooled cause-specific survivals after multiple imputation were satisfactory, the older the patients, the worse the survival. In contrast, net survival results were questionable, with a net survival of 105 % at 10 years for older patients. Again, the pooled cause-specific survival using competing risks method was almost equal to that of Kaplan-Meier estimator.

**Table 6:** Overall survival, net survival and cause-specific survivals of Kaplan-Meier and competing risks after multiple imputation with their confidence intervals according to age

		Overall	Net	Pooled Kaplan-Meier	Pooled competing risks
[56;65]	1 year	0.99 [0.99-1]	1 [0.99-1.01]	0.996 [0.989-0.999]	0.996 [0.989-0.999]
	3 years	0.96 [0.95-0.97]	0.99 [0.98-1.01]	0.983 [0.972-0.991]	0.983 [0.972-0.991]
N=867	5 years	0.93 [0.91-0.95]	0.99 [0.98-1.02]	0.977 [0.963-0.987]	0.977 [0.964-0.987]
	10 years	0.86 [0.82-0.9]	0.99 [0.94-1.04]	0.951 [0.917-0.974]	0.953 [0.922-0.974]
[65;70]	1 year	0.99 [0.99-1]	1.01 [1.01-1.02]	0.997 [0.991-0.999]	0.997 [0.991-0.999]
	3 years	0.93 [0.92-0.95]	0.99 [0.98-1.01]	0.976 [0.964-0.985]	0.976 [0.964-0.985]
N=1064	5 years	0.89 [0.87-0.91]	0.98 [0.96-1]	0.955 [0.938-0.969]	0.957 [0.940-0.970]
	10 years	0.79 [0.75-0.84]	0.99 [0.95-1.05]	0.933 [0.897-0.960]	0.937 [0.905-0.962]
[70;++]	1 year	0.97 [0.96-0.98]	1 [0.99-1.01]	0.992 [0.983-0.996]	0.992 [0.984-0.996]
	3 years	0.91 [0.90-0.94]	0.99 [0.98-1.01]	0.966 [0.951-0.977]	0.967 [0.953-0.978]
N=913	5 years	0.85 [0.82-0.88]	0.99 [0.96-1.02]	0.952 [0.931-0.969]	0.954 [0.934-0.970]
	10 years	0.76 [0.70-0.83]	1.05 [0.97-1.14]	0.926 [0.871-0.963]	0.931 [0.884-0.964]

### Sensitivity analysis for multiple imputation method

In the previous paragraph, we have seen that the pooled cause-specific survivals of Kaplan-Meier and competing risks were nearly equal. In this section, only Kaplan-Meier cause-specific survival was performed to test the sensibility of our multiple imputation model.

#### First method

**Fig. 2:**
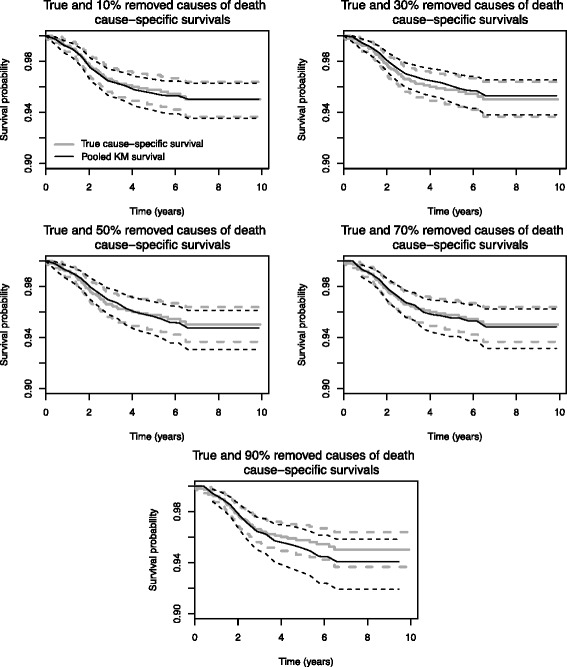
True cause-specific, pooled cause specific survival rates and their confidence intervals using the first sensibility method

As can be seen in Fig. 2, the true cause-specific survival curve and the pooled cause-specific survival curve were confounded for up to 70 % of removed causes of death. For 90 % removed causes of death, the pooled cause-specific survival underestimated the true cause-specific survival, but this remained in the confidence interval of the latter.

Thus, with our 50 % of missing causes of death, and our imputation model, it appears that we obtained a good estimation of the cause-specific survival.

#### Second method

**Fig. 3:**
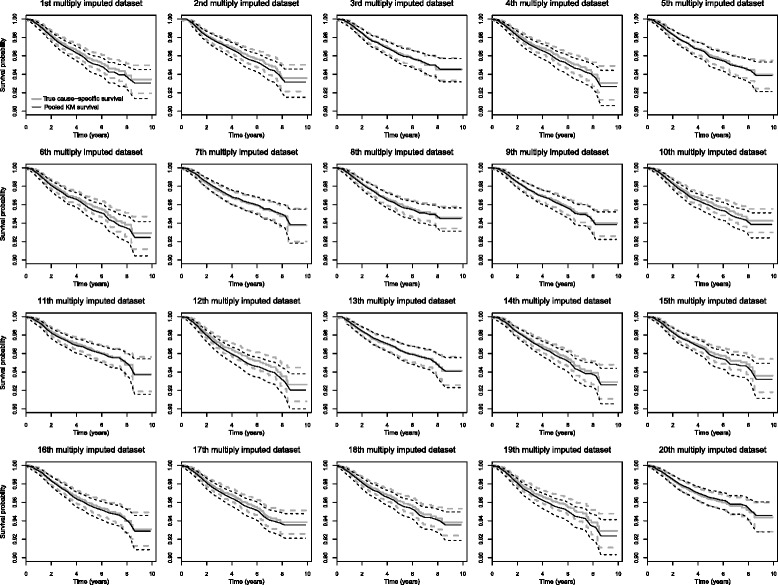
True cause-specific, pooled cause-specific survival rates and their confidence intervals using the second sensibility method

Figure 3 shows the true and the pooled cause-specific survival rates estimated for each of the 20 first multiply imputed datasets. The survival estimates provided by our imputation model were in agreement with the true cause-specific survival.

#### Third method

**Fig. 4:**
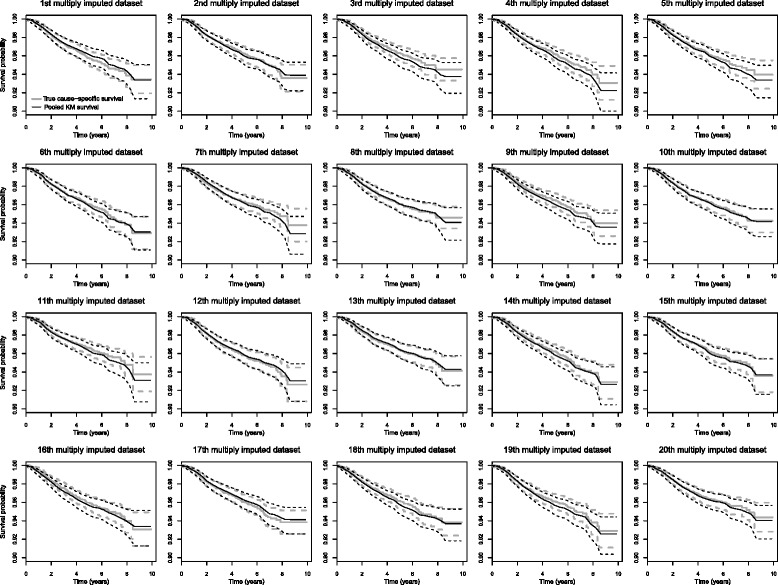
True cause-specific and pooled cause-specific survival rates and their confidence intervals using the third sensibility method

The results are displayed in Fig. 4. The results were satisfactory. Indeed, the true cause-specific survival always remained in the confidence interval of the pooled cause-specific survival.

## Discussion

On the basis of prostate cancer data, we estimated the two pooled cause-specific survival rate using Kaplan-Meier’s [18] estimator and competing risks after multiple imputation, as well as the net survival rate using Pohar-Perme’s estimator [5].

By definition, net survival presupposes that prostate cancer was the only cause of death. When considering cause-specific survival of Kaplan-Meier, the event is death by prostate cancer, and death by other causes is censored. Causes of death are not required when estimating net survival, which is a very useful method, but, conversely, based on the general population mortality rate. Since Kaplan-Meier estimates can only be interpreted as estimates of net survival if it is reasonable to assume independence between prostate cancer death and death from other causes, we also estimated cause-specific cumulative incidence estimator, accounting for deaths due to other causes as competing risks. Survivals were almost equal, so it was reasonable to assume independence between prostate cancer death and death from other causes.

This work has some limitations. Assuming that the assumptions of the models were verified, Pohar-Perme’s and Kaplan-Meier’s estimators should theoretically estimate the same quantity; however, we showed that this was not the case, probably because men presenting with prostate cancer are not representative of men presenting with a cancer in the general population.

Cause-specific survival appeared as more precise, but obtaining the causes of death is very difficult in practice; it assumes that the causes of death are accurate whereas it is very difficult to gather all the causes of death in a large sample.

Therefore, we used the multiple imputation method to overcome this difficulty and calculated the cause-specific survival. Our results are satisfactory even when applying a 50 % missing rate for causes of death, because the MAR hypothesis holds for the variable causes of death. Moreover, with the first method of sensitivity analysis, on the sub database of 1422 patients, the multiple imputation model correctly estimated the missing causes of death as up to 50 % missing and the results of the Gelman’s cross-validation [42] validated our imputation model.

However, depending on the objectives, this method may not be applicable to very large databases for example, since the multiple imputation method is demanding in terms of time resources. Nevertheless, it is affordable when databases are not too large.

## Conclusion

In our data set, the results obtained by multiple imputation appeared to be better and more realistic than those obtained using the net survival rate. Thus, we wonder whether it would perhaps be more efficient to use multiple imputation first, rather than net survival, when a representative subsample of causes of death being completed is validated by experts.

## Additional files

Additional file 1
**Description of d’Amico score.** (PDF 36 kb)

Additional file 2
**Mean and standard deviation of the synthetic values plotted against iteration for the imputed data.** Treat1: first treatment; PSAlevel: PSA at diagnostic and PSA.post: PSA after treatment. (PDF 38 kb)

## Abbreviations

ERSPC: European Randomized Study of Screening for Prostate Cancer
MICE: multiple imputation by chained equation
HIFU: High intensity focused ultrasound
PSA: Prostatic specific antigens
RNIPP: Répertoire national d’identification des personnes physiques
CépiDC: Centre d’ épidémiologie sur les causes médicales de décès
MCAR: Missing completely at random
MAR: Missing at random
MNAR: Missing not at random
