# Supplementary material for: Prostate cancer: net survival and cause-specific survival rates after multiple imputation
Source: BMC Med Res Methodol. 2015 Jul 28;15:54. doi: 10.1186/s12874-015-0048-4 (PMC4517373; doi:10.1186/s12874-015-0048-4)
Supplement: Additional file 1 — Description of d’Amico score. (PDF 36 kb) [file 12874_2015_48_MOESM1_ESM.pdf]

Description of d'Amico score.

d'Amico generalized	Description
Localized prostate cancer with low risk	cT = 1a,1b,1c or 2a<br>and Gleason ≤ 6<br>and PSA at diagnosis ≤ 10 ng/ml
Localized prostate cancer with intermediate risk	cT = 2b<br>or Gleason = 7<br>or $10 <$ PSA at diag ≤ 20 ng/ml
Localized prostate cancer with high risk or locally advanced	cT = 2c, 3a, 3b or 4<br>or Gleason > 7<br>or PSA at diag > 20 ng/ml
Prostate cancer with invaded lymph node or metastatic	cN =1 or cM = 1
