# Supplementary figures and images for: Prostate cancer: net survival and cause-specific survival rates after multiple imputation
Source: BMC Med Res Methodol. 2015 Jul 28;15:54. doi: 10.1186/s12874-015-0048-4 (PMC4517373; doi:10.1186/s12874-015-0048-4)

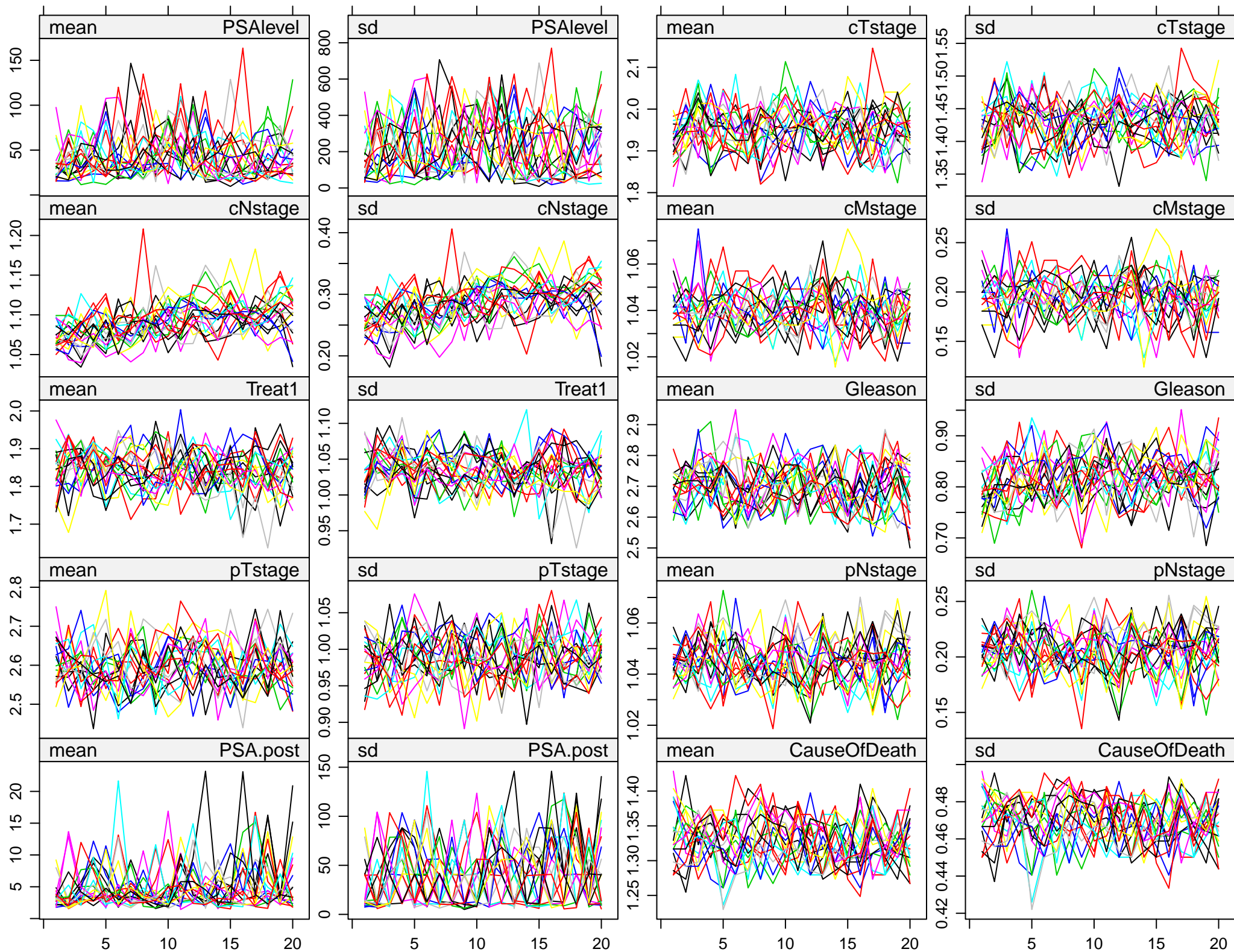

Iteration

Supplement: Additional file 2 — Mean and standard deviation of the synthetic values plotted against iteration for the imputed data. Treat1: first treatment; PSAlevel: PSA at diagnostic and PSA.post: PSA after treatment. (PDF 38 kb) [file 12874_2015_48_MOESM2_ESM.pdf]
